# Identification of a *HOXD13* variant in a Mongolian family with incomplete penetrance syndactyly by exon sequencing

**DOI:** 10.1186/s12920-022-01360-3

**Published:** 2022-10-04

**Authors:** Husile Husile, Zhifeng Wu, Liqing Yang, Yaning Cao, Qizhu Wu

**Affiliations:** 1 Affiliated Hospital of Inner Mongolia Minzu University, 028000 Tongliao, China; 2 Inner Mongolia Engineneering and Technical Research Center for personalized Medicine, 028000 Tongliao, China; 3 College of Life Sciences and Food Engineering, Inner Mongolia Minzu University, 028000 Tongliao, China; 4 School Of Life Sciences, Inner Mongolia University, 010000 Hohhot, China

**Keywords:** Family, Syndactyly, *HOXD13* gene

## Abstract

**Background:**

Syndactyly (SD) refers to a deformity caused by the fusion and limb differentiation disorder of soft tissues and/or skeletons to varying extents between adjacent fingers (toes). The main features of this disease are phenotypic heterogeneity and genetic heterogeneity. In this study, we examined four generations of a Chinese Mongolian with different phenotypes of syndactylia and analysed and identified the pathogenic genetic variants of SD by exon sequencing.

**Methods:**

The clinical phenotypes of patients were analysed, and the hands and feet were examined by X-ray. The pedigree was drawn, and the family data were analysed. Peripheral blood was collected from the family members, and genomic DNA was extracted. The candidate genes of SD were identified by exon sequencing, and the mutation sites of the captured candidate genes were amplified by PCR and verified by Sanger sequencing.

**Results:**

The family has congenital syndactyly, which is an autosomal dominant disease. At present, this condition has been passed down for 4 generations and was identified in 9 patients, including 4 males and 5 females. Five patients, I_2_, II_4_, III_5_, III_,7_ and III_10_, had unilateral syndactyly, and four patients, III_16_, IV_3_, IV_6_ and IV_7_, had bilateral finger syndactyly. All of their toes were unaffected. The proband and the other patients in this family had a c.917G > A (p.R306Q) mutation, which is located at position 917 of the second exon of the *HOXD13* gene. This mutation results in a change in the amino acid at position 306, in which arginine is changed to glutamine. This mutation cosegregates in unaffected individuals and affected patients in this family. Moreover, 201 Mongolian genome databases and a thousand human genome databases were referenced to further confirm that the pathogenic genetic variant that causes syndactyly in this family is found in *HOXD13*.

**Conclusion:**

This study found that the mutation site of the pathogenic gene in this family was *HOXD13*, c.917G > A (p.R306Q). The phenotype of the family member III_12_ was normal, but this member was also a carrier of the pathogenic genetic variant. This indicates that the disease of this family has incomplete penetrance characteristics. Our results further enrich the expression profile of the *HOXD13* gene.

**Supplementary Information:**

The online version contains supplementary material available at 10.1186/s12920-022-01360-3.

## Background

Syndactyly (SD) refers to the deformity of partial or complete fusion of adjacent fingers or toes caused by abnormal influencing factors during limb development. SD has obvious phenotypic heterogeneity and genetic heterogeneity, and SD is characterized by being symmetric or asymmetric and unilateral or bilateral. The complex clinical phenotype of SD leads to different degrees of deformity, which can manifest as abnormal skin texture or involve phalanges, metacarpals or metatarsals and more seriously extend to carpal or tarsal bones [1]. At present, 9 kinds of nonsyndromic SD (type I-IX) have been reported. Among these 9 types, the five main types (I-V type) are autosomal dominant inheritance, and the other types and subtypes are inherited in an autosomal recessive or X‑linked hereditary pattern [2]. Syndactyly type I is the most common type, and it can manifest as centre axis webbing of 3/4 syndactyly and/or 2/3 toe syndactyly. According to the phenotype and genotype, this type can be divided into four subtypes (type I-a, type I-b, type I-c, and type I-d). Syndactyly I-c (Montagu) is associated with skin/bone fusion of only the third and fourth fingers and occasionally the third to fifth fingers [3]. In the process of limb development, the signalling and transcriptional regulation of genes are involved in this highly complex growth process. It is known that the genes *HOXD13*, *SHH*, *GLI3*, *GJA1*, *LRP4*, *WNT7A* and *APC* play an important role in limb development. Different mutations of these genes may lead to limb deformities. It has been reported that deletions, nonsense mutations, missense mutations, frameshift mutations or polyalanine expansion mutations of the *HOXD13* gene could lead to SD [4, 5]. In this study, we examined a four-generation Mongolian family with congenital syndactyly from Eastern Inner Mongolia, China. To identify the pathogenic genetic variants of this family, we used exon sequencing analysis and related analysis methods to research this Mongolian family.

## Methods

### Clinical data collection

**Fig. 1:**
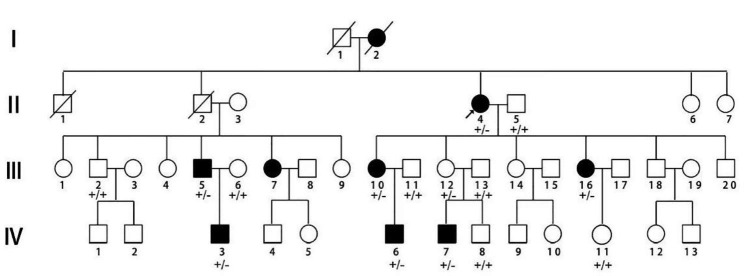
A pedigree with syndactyly ■and●represent male patients and female patients respectively;□and○represent normal men and normal women respectively;↗represents the proband (II4);+/−: heterozygous for *HOXD13*, +/+: wild type for *HOXD13*.

**Fig. 2:**
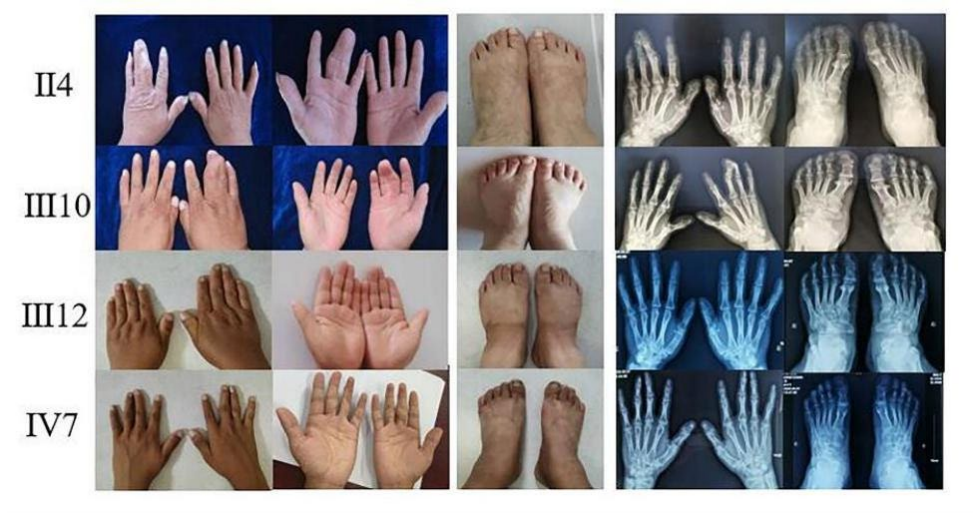
Pictures and X-rays of syndactyly of pedigree patients II4 (proband)the third and fourth fingers of the left hand were combined, and the toes were normal; III10 (patient)the third and fourth fingers of the right hand were combined, and the toes were normal; III12 Normal phenotype but carrier of pathogenic gene; IV7 third and fourth fingers of both hands were combined, and two repair operations have been performed.

A Mongolian family with congenital syndactyly in eastern Inner Mongolia was selected as the research object. The proband (II_4_) in this family was a 65-year-old female. Genetic counselling was conducted at the Affiliated Hospital of Inner Mongolia Minzu University for the first time in March 2014. This condition has been passed down for 4 generations in this family. The medical history of 38 members of this family can be traced back by investigation (Fig. 1), and 9 patients with this condition were identified (4 males and 5 females). III_16_ underwent finger separation surgery with the traditional folk method in infancy and had a poor prognosis. Two patients, IV_6_ and IV_7_, underwent operations in the Department of Hand and Foot Surgery of the Affiliated Hospital of Inner Mongolia Minzu University between the ages of 5 and 12, and the prognosis was good (Table 1). The hand pictures and X-ray results of II_4_, III_10_, III_12_ and IV_7_ are shown in Fig. 2.

All study protocols were approved by the Ethics Committee of the Affiliated Hospital of Inner Mongolia Minzu University. The ethical approval ID is NM-LL-2018-03-03-01. All experimental work was performed in accordance with the Declaration of Helsinki. Informed consent forms were obtained from all the participants for genetic analysis of the DNA samples and publication of the genetic data. We collected basic information, medical histories and X-ray results. To date, the clinical data of 15 members have been collected. The amount of blood collected was 10ml, and it was stored in two EDTA anticoagulant tubes for further study.

**Table 1:** Clinical characteristics of the family

Family ID	Gender	Years	Deformities in hands	Side of affected hand	Operation
II4	Female	65	Unilateral 3/4 finger fusion	Left	-
III5	Male	50	Unilateral 3/4 finger fusion	Right	-
III7	Female	53	Unilateral 3/4 finger fusion	Left	-
III10	Female	45	Unilateral 3/4 finger fusion	Right	-
III16	Female	39	Bilateral 3/4 finger fusion	Left and right	Corrective surgery for both hands
IV3	Male	27	Bilateral 3/4 finger fusion	Left and right	-
IV6	Male	19	Bilateral 3/4 finger fusion	Left and right	Corrective surgery for both hands
IV7	Male	17	Bilateral 3/4 finger fusion	Left and right	Correction and repair of both hands

## Extraction of genomic DNA

We collected 5ml peripheral blood from family volunteers with EDTA anticoagulant. Blood genomic DNA was extracted by protease K-SDS-phenol/chloroform extraction. Extracted DNA was quantified using a Qubit 3.0 Fluorometer. Agarose gel electrophoresis was used to analyse the extent of DNA degradation and to assess whether there was contamination of RNA and protein.

## Exon sequencing and data analysis

In this experiment, six members of the family (three patients, II_4_, III_10_ and IV_7_, and two unaffected members, III_2_, III_13_ and III_12_) were selected for whole exon sequencing to capture the candidate genes. Exon sequencing was completed by Novo gene.

**Fig. 3:**
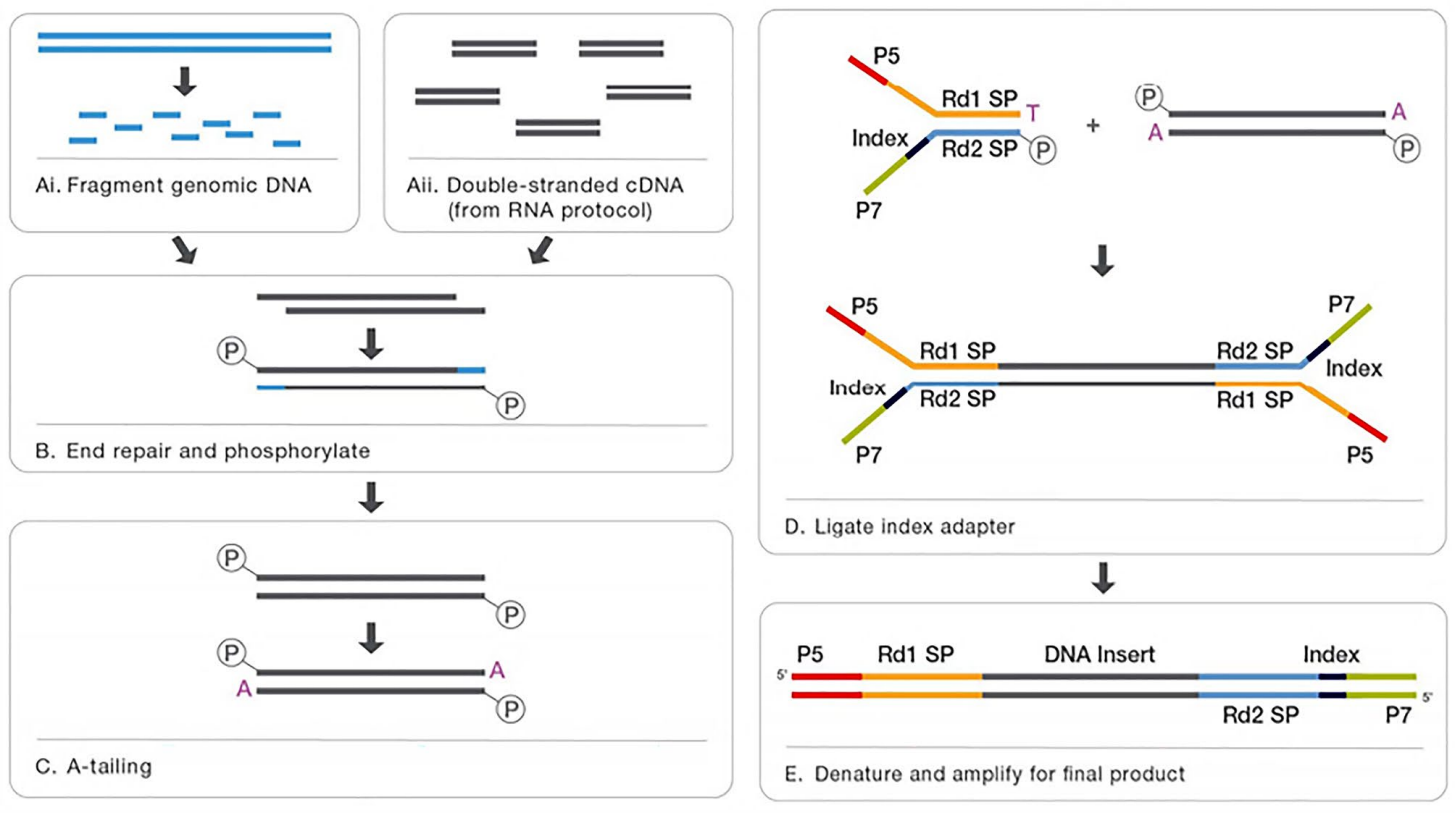
Library construction and exon capture The genomic DNA was randomly broken into segments with a growth of 180-280 bp.After end repair and adding A tail, connectors were connected at both ends of the fragment to prepare DNA library. The library with specific index was pooled and hybridized with biotin labeled probe in liquid phase, and then the exons on the gene were captured.

The operation process is reported here. After extracting genomic DNA, the library was constructed, and the exons of the library products were captured. In the capture process, an Agilent liquid-phase probe capture system was used to efficiently enrich human DNA in the whole exon region. After PCR amplification, library quality inspection was carried out, and Illumina NovaSeq 6000 PE150 sequencing (pe150, pair end 150 bp, which means it is high-throughput two terminal sequencing with 150 bp measured at each end) was carried out according to the effective concentration of the qualified library and the data output requirements (Fig. 3). The sequencing depth was 100x, and the original sequence (raw reads) was obtained by sequencing. To guarantee the quality of information analysis, raw reads were finely filtered to obtain clean reads, and the subsequent analysis is based on the clean reads. The specific process involved the removal of reads paired with the adapter and the removal of reads paired with a proportion of N (N indicates that the base information cannot be determined) greater than 10% in the single-end sequencing read. When the base number of low-quality (less than 5) bases contained in the single-end sequencing read exceeds 50% of the length ratio of the read, this pair of reads was removed. This ensures that the quality of the sequencing data is mainly distributed over Q30 (≥ 80%), and the remaining valid data are clean reads.

Clean reads were compared to the reference genome (GRCh37/hg19) through BWA software. The initial comparison results in BAM format were obtained and then sorted with SAMtools. Sambamba marks repeated reads (marked duplicate reads) and identifies SNP sites through Sentieon. The conversion/transversion ratio (Ts:Tv) can reflect the accuracy of the SNP datasets, and this ratio in the whole genome was approximately 2.2. ANNOVAR software was used to annotate SNPs, including the annotation information of the dbSNP database, thousand human genome project database and other existing databases. CNVkit software was used to detect CNVs. CNVs were detected by determining the depth distribution of reads on a reference genome. ANNOVAR software was used to annotate the copy number variations. Finally, candidate genes were screened through annotation.

## PCR amplification and Sanger sequencing validation

Primer design, PCR amplification and Sanger sequencing were carried out for the six candidate genes obtained by exon sequencing. The primers for PCR amplification of the discovered pathogenic variant were as follows: *HOXD13* forward primer, 5’-CTGCACCCCTGCAAACGCAC-3’; and *HOXD13* reverse primer, 5’-GCTGTCTGTGGCCAACCTGG-3’. The PCR amplification reaction conditions were as follows: 3 min predenaturation at 95 °C; 30 s denaturation at 94 °C, 30 s annealing at 55 °C, and 45 s extension at 72 °C for 30 cycles; and 72 °C for 5 min. The primer sequences and amplification conditions of the *FBN2*, *FMN1*, *FREM2*, *LRP4* and *MYO10* genes are indicated in Additional file 1: Table S1.

The PCR products were detected by 2% agarose gel electrophoresis, recovered from the gel (TIAN gel Midi Purification Kit), and sent to Beijing Liuhe Huada Gene Technology Co Ltd. for Sanger sequencing.

## Results

### Phenotypic characteristics

This Mongolian family included 38 members that spanned 4 generations. There were 9 patients involved, including 4 males and 5 females. Patients appear in each generation. The incidence probability for men and women is equal, which reflects an autosomal dominant inheritance model. I_2_, II_4_, III_5_, III_7_ and III_10_ were unilateral 3/4 finger fusions, and all of these patients had normal toes. III_16_, IV_3_, IV_,6_ and IV_,7_ had bilateral 3/4 finger fusion, and all of these patients had normal toes. No other diseases were identified for the 9 patients and other major family members who underwent a systematic physical examination. The mother (III_12_) of patient IV_7_ had no clinical phenotype, but the test results showed that she was a carrier of pathogenic genes.

## Exon sequencing results

Exon sequencing for performed for six family members, including the proband (3 patients, 1 suspected pathogenic gene carrier and 2 unaffected members), and a total of 6 candidate genes related to finger (toe) development were screened, namely, the *FBN2, FMN1, FREM2, HOXD13, LRP4* and *MYO10* genes (Table 2).

**Table 2:** Genetic information

Gene	Chromosome position	SNP	Mutation information
*HOXD13*	chr2(q31.1)	rs879255265	G > A
*FBN2*	chr5(q23.3)	rs32209	T > C
*MYO10*	chr5(p15.1)	rs11750538	G > A, T
*LRP4*	chr11(p11.2)	rs3816614	C > A, T
*FREM2*	chr13(q13.3)	rs2496425	T > A, C
*FMN1*	chr15(q13.3)	rs1399078	A > C

## Sanger sequencing results

**Fig. 4:**
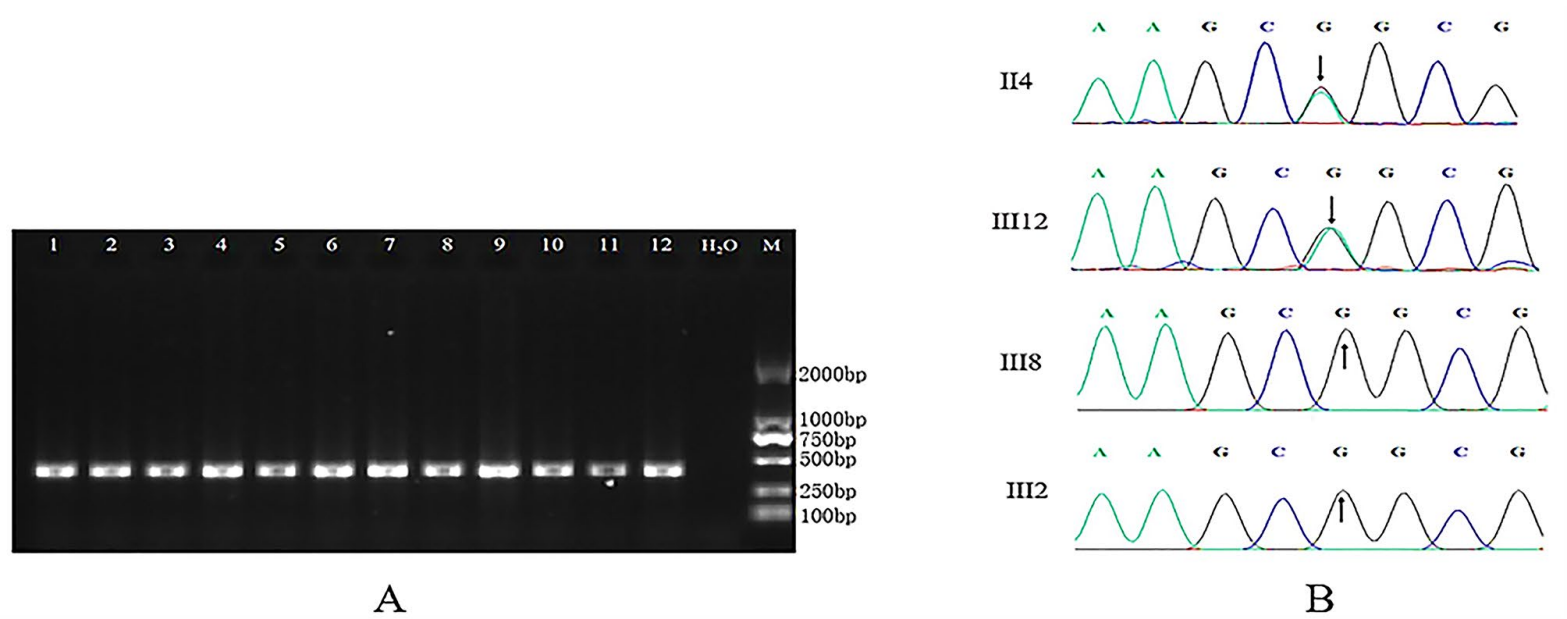
Verification results (*HOXD13*) A.2%agarosegel electrophoresis was used to detect PCR products: subjects; H2O.Negative control; M. DL 2000 DNA marker (Note: the gel figure was cropped; The original gel image is in Additional file3: Figure S2). B. Sanger sequencing validation results: II4 proband is GA heterozygous; The carriers with incomplete penetrance of III12 has normal phenotype, but the genotype is GA heterozygous; III8 normal member, genotype is GG homozygous; III2 normal member, the genotype is GG homozygous.

The *HOXD13*, *FBN2*, *FMN1*, *FREM2*, *LRP4* and *MYO10* genes were amplified by PCR. All samples contained the target DNA fragments (target fragment lengths were 388 bp for *HOXD13*, 445 bp for *FBN2*, 382 bp for *FMN1*, 688 bp for *FREM2*, 764 bp for *LRP4*, and 685 bp for *MYO10*). Further Sanger sequencing validation showed that in this family, the patient and the carrier without clinical phenotype (III_12_) had a G > A mutation in the second exon at position 917 of the *HOXD13* gene (*HOXD13*, c.917G > A) (Fig. 4) that resulted in the change of arginine at position 306 to glutamine. This mutation was not seen in the unaffected family members, indicating that this site has a cosegregation phenomenon between patients and unaffected members of this family. In addition, the mutation sites of the *FBN2*, *MYO10*, *LRP4*, *FREM2* and *FMN1* genes were not cosegregated in the patients and unaffected members of this family (Additional file 2: Figure S1). Moreover, referring to the genomes of 1000 people and 201 Mongolian genome databases, this *HOXD13* gene mutation (c.917G > A) was not found, indicating that the *HOXD13* gene c.917G > A, p.R306Q mutation is the main cause of disease in this Mongolian family.

## HOXD13 function prediction analysis

**Fig. 5:**
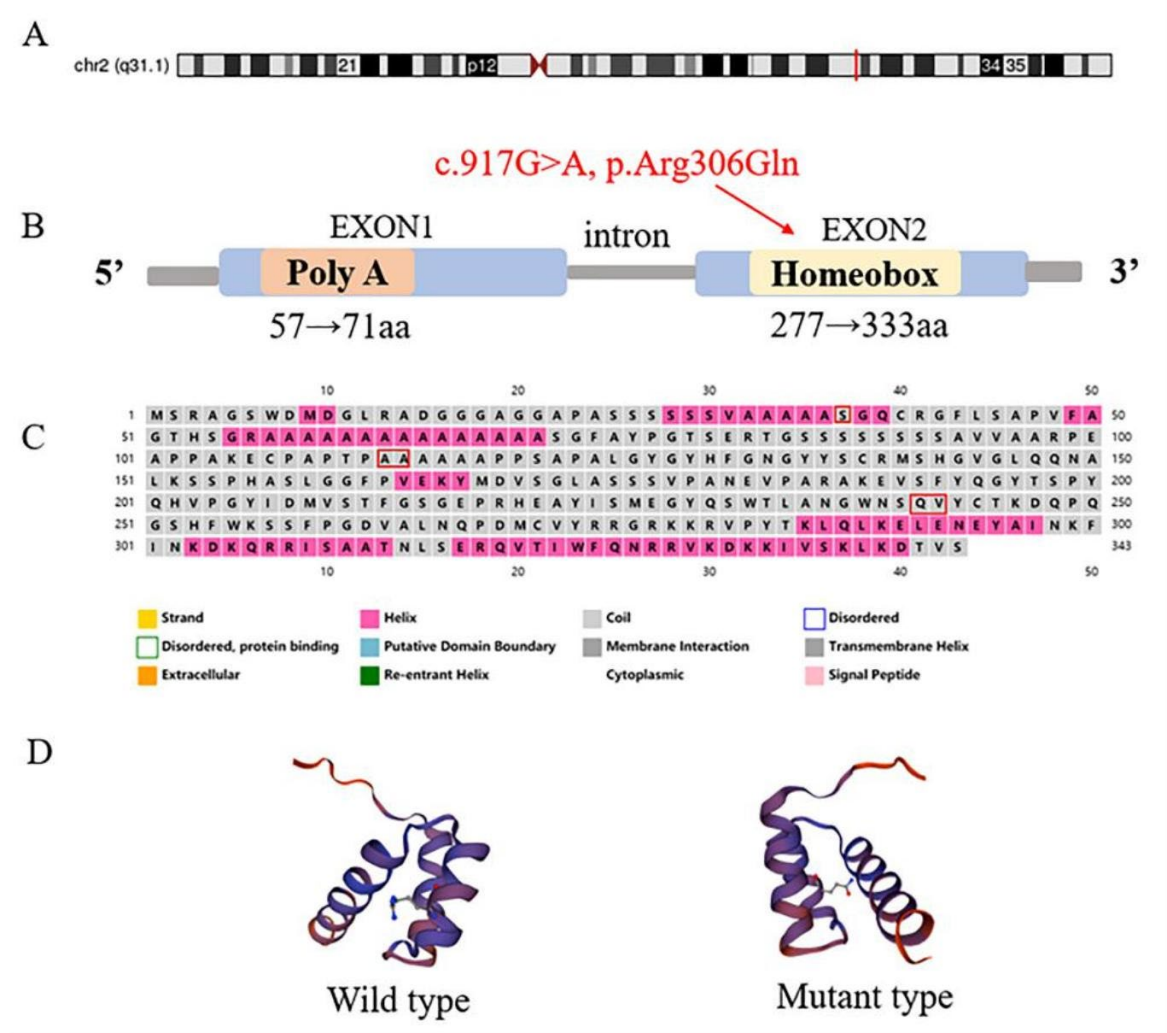
Function prediction of *HOXD13* gene mutation (A) *HOXD13* is located on chromosome 2 q31.1 (red bar). (B) The gene structure of the *HOXD13*. (C) Sequence Plot. (D) Protein structure of HOXD13 wild type and mutant type.

The *HOXD13* gene is a member of the Hox gene family and is located at 2q31.1. It contains two exons and is 1365 bp in length. It encodes a 343 amino acid polypeptide sequence. The *HOXD13* gene c.917G > A mutation is located in exon 2, which encodes the homeodomain in the C-terminal region (a highly conserved DNA binding motif). A protein secondary structure prediction website (http://bioinf.cs.ucl.ac.uk/psipred/) was used to predict the structure of the mutated sequence. The results show that amino acids at the 30th ~ 40th, 110th ~ 120th and 240th ~ 250th positions composing the alpha helix form an irregular curl. The homologous protein sequence for homebox protein Hox-D13 [Homo sapiens] was obtained from NCBI (National Center for Biotechnology Information). The protein three-dimensional structure prediction website SWISS-MODEL (www.swissmodel.expasy.org/) was used to predict the protein structure. The mutation of the *HOXD13* gene c.917G > A, p.R306Q changes the main chemical bonds that maintain the three-dimensional structure of the protein (Fig. 5).

## Discussion

According to the data of Online Mendelian Inheritance in Man (OMIM), 287 gene mutations have been associated with syndactyly. In this study, six candidate genes related to finger (toe) development were screened by exon sequencing: the *FBN2*, *FMN1*, *FREM2*, *HOXD13*, *LRP4* and *MYO10* genes. The variation sites of the *FBN2* gene are mainly missense variations and nonsense variations, and these variations are an important cause of congenital contractile arachnodactyly (CCA). The main manifestation of this condition is that patients usually have permanent joint curvature (contracture), such as finger and toe curvature (toe flexion) [6, 7]. The Cenani-Lenz syndrome phenotype is related to various mutations of the *LRP4* gene. Its potential mechanism of action may be involved in the impaired membrane transport of LRP4 protein [8, 9]. The rearrangement of the *GREM1-FMN1* gene and *FMN1* mutations and abnormal limb malformations, such as heart malformation, cartilage hypoplasia and polydactyly, are associated with the loss of FMN1 protein [10]. A study of Fraser syndrome in a foetus found that mutations of *FREM2* may lead to symptoms of double fingers and hidden eyeballs in foetuses with congenital high airway obstruction syndrome and renal hypoplasia after birth. However, abnormalities in the fused eyelids and fingers, larynx and vagina are caused by failed programmed cell death [11, 12]. The *MYO10* gene plays a certain role in limb development and promotes cell migration in vivo and in vitro [13, 14]. The six candidate genes obtained by second-generation sequencing in this study were analysed by Sanger sequencing and other methods. The results showed that there was no cosegregation of *FBN2*, *FMN1*, *FREM2*, *LRP4*, *MYO10* or other gene mutations in patients and unaffected members of this family, so these genes were excluded as the main pathogenic genetic variants of this family.

Clinically, *HOXD13* gene mutations have the strongest correlation with familial syndactyly. According to the mutation location and type, mutations can be divided into functional mutations, such as alanine chain length variations, homologous box amino acid variations, and splice site mutations [15]. The *HOXD13* gene has two exons. Exon 1 encodes a 15 residue polyalanine region of the *HOXD13* N-terminal region. Mutations of the homologous box amino acid encoded by exon 2 can reduce the DNA binding activity of *HOXD13* or change the recognition of its target gene. This results in a multi finger (toe) or short finger (toe) phenotype [16].

Through genetic research involving several SD families, it was found that syndactyly type I-c, SPD1 (synpolydactyly type 1) and syndactyly type V (SDTY5; MIM# 186,300) were caused by mutations in homeobox D13 (HOXD13) and the c. 917G > A (p.R306Q) mutant affects the ability of *HOXD13* to activate transcription. Mutations in the HOXD13 homologous domain (p.R306Q) can cause SD1-c, and there is a similarity between SD1-c and polydactyly syndrome [17, 18]. Another study reported that an SPD deformity caused by a missense mutation of the *HOXD13* gene homeodomain was found in a Chinese family [19]. There are also studies on the mutation of the *HOXD13* gene in an SPD family, which further confirms that PAE mutations in the *HOXD13* gene in the Chinese population can lead to typical SPD [20]. Studies have reported that the *HOXD13* gene has different genetic defects in mouse models simulating human multi finger and toe phenotypes [21]. These results further support the importance of the *HOXD13* gene in limb development.

SPD1 is characterized by syndactyly of the middle axis (3/4 fingers) and polydactyly of the posterior axis (4/5 toes). Type V syndactyly showed 4/5 syndactyly with metacarpal fusion, 4/5 metacarpal dysplasia and axial webbing. The clinical features of type I-c syndactyly are only 3/4 fingers, skin/bone fusion and normal toes [1, 2].

In the Mongolian family of this study, patients I_2_, II_4_, III_5_, III_,7_ and III_10_ had unilateral 3/4 finger fusion, III_16_, IV_3_, IV_6_ and IV_7_ had bilateral 3/4 finger fusion, and no toes were affected. This indicates type I-c syndactyly. The mother (III_12_) of patient IV_7_ had no clinical phenotype, but the test results showed that she was a carrier of a pathogenic gene. II_2_ in the pedigree died. According to the relatives’ description, this individual was asymptomatic, and their children (III_5_ and III_7_) were affected. This indicates that the family has the characteristics of incomplete penetrance. In addition, the patients in this family had inconsistent presentations. Some had unilateral hand involvement, and some had bilateral hand involvement. Furthermore, the phenomenon was gradually aggravated with transmission from generation to generation. These complex clinical phenotypes may be related to gene interactions and genetic backgrounds, and these considerations need to be further studied.

## Conclusion

Exon sequencing was used to further verify that there was a G > A mutation (*HOXD13*, c.917G > A, p.R306Q) in the second exon at position 917 of the *HOXD13* gene in patients and carriers of this family. This mutation resulted in the change of arginine at position 306 to glutamine. This mutation could lead to the occurrence of syndactyly, and it is the main cause syndactyly in this Mongolian family. Our discovery also enriched the expression profile of the *HOXD13* gene.

## Electronic supplementary material

Below is the link to the electronic supplementary material.

Supplementary Material 1

Supplementary Material 2

Supplementary Material 3

## Data Availability

The datasets generated during the current study are not publicly available because it is possible that individual privacy could be compromised, but are available from the corresponding author on reasonable request.

## Abbreviations

SD: Syndactyly.
SPD1: Synpolydactyly type1.

## References

[1] Chouairi F, Mercier MR, Persing JS, et al. National Patterns in Surgical Management of Syndactyly: A Review of 956 Cases. Hand (N Y).2020;15(5):666–673. 10.1177/1558944719828003 PMC7543215 30770023

[2] Malik S (2012). Syndactyly: phenotypes, genetics and current classification. Eur J Hum Genet.

[3] Ahmed H, Akbari H, Emami A (2017). Genetic overview of syndactyly and polydactyly. Plast Reconstr Surg Glob Open.

[4] TahirZaib WeiJi (2019). A heterozygous duplication variant of the *HOXD13* gene caused synpolydactyly type 1 with variable expressivity in a Chinese family. BMC Med Genet.

[5] Guo R, Fang X, Mao H (2021). A Novel Missense Variant of *HOXD13* Caused Atypical Synpolydactyly by Impairing the Downstream Gene Expression and Literature Review for Genotype–Phenotype Correlations. Front Genet.

[6] Guo X, Song C, Shi Y (2016). Whole exome sequencing identifies a novel missense *FBN2* mutation co-segregating in a four-generation Chinese family with congenital contractural arachnodactyly. BMC Med Genet.

[7] Guoling You B, Zu B, Wang (2017). Exome Sequencing Identified a Novel *FBN2* Mutation in a Chinese Family with Congenital Contractural Arachnodactyly. Int J Mol Sci.

[8] Igor Fijalkowski E, Geets E, Steenackers (2016). A Novel Domain-Specific Mutation in a Sclerosteosis Patient Suggests a Role of *LRP4* as an Anchor for Sclerostin in Human Bone. J Bone Miner Res.

[9] Nuha Alrayes A, Aziz F, Ullah (2020). Novel missense alteration in *LRP4* gene underlies Cenani-Lenz syndactyly syndrome in a consanguineous family. J Gene Med.

[10] Dimitrov BI, Voet T, De Smet L (2010). Genomic rearrangements of the *GREM1-FMN1* locus cause oligosyndactyly, radio-ulnar synostosis, hearing loss, renal defects syndrome and Cenani–Lenz-like non-syndromic oligosyndactyly. J Med Genet.

[11] Bouaoud J (2020). Fraser syndrome: review of the literature illustrated by a hystorical adult case. Int J Oral Maxillofac Surg.

[12] Shoko, Ikeda (2020). Prenatal diagnosis of Fraser syndrome caused by novel variants of *FREM2*. Hum Genome Var.

[13] Doreen Becker (2014). A variant in *MYO10* is associated with hind limb conformation in Swiss Large White boars. Anim Genet.

[14] He J-H, Chen J-G, Zhang B (2020). Elevated *MYO10* Predicts Poor Prognosis and its Deletion Hampers Proliferation and Migration Potentials of Cells Through Rewiring PI3K/Akt Signaling in Cervical Cancer. Technol Cancer Res Treat.

[15] WangB XuB (2012). A novel non-synonymous mutation in the homeodomain of *HOXD13* causes synpolydactyly in a Chinese family. Clin Chim Acta.

[16] Nathalie Brison P, Debeer P, Tylzanowski (2014). Joining the fingers: A *HOXD13* story. Dev Dyn.

[17] DaiL, LiuD SongM (2014). Mutations in the homeodomain of *HOXD13* cause syndactyly type 1-c in two Chinese families. PLoS ONE.

[18] Tahir Zaib H, Rashid H, Khan (2022). Recent Advances in Syndactyly: Basis, Current Status and Future Perspectives. Genes (Basel).

[19] Radhakrishnan P, Nayak SS, Pai MV (2017). Occurrence of Synpolydactyly and Omphalocele in a Fetus with a *HOXD13* Mutation. J Pediatr Genet.

[20] Brison N, Debeer P, Fantini S (2012). An N-terminal G11A mutation in *HOXD13* causes synpolydactyly and interferes with Gli3R function during limb pre-patterning. Hum Mol Genet.

[21] Hao Deng T, Tan Q, He (2017). Identification of a missense *HOXD13* mutation in a Chinese family with syndactyly type I-c using exome sequencing. Mol Med Rep.

