# Supplementary material for: Identification of a HOXD13 variant in a Mongolian family with incomplete penetrance syndactyly by exon sequencing
Source: BMC Med Genomics. 2022 Oct 4;15:210. doi: 10.1186/s12920-022-01360-3 (PMC9533607; doi:10.1186/s12920-022-01360-3)
Supplement: Supplementary file 1 — Supplementary Material 1 [file 12920_2022_1360_MOESM1_ESM.pdf]

Additional file 1

Primer sequences and amplification conditions of *FBN2*, *FMN1*, *FREM2*, *LRP4* and *MYO10* genes

Table S1 Gene primer sequence

Primer name	Primer sequence	Fragment length (bp)	Annealing temperature (℃)
FBN209	F: TGGAGGCATTACATAAGC	445	55
FBN209	R: CCGAGATAAGATGGGTTG		
MYO1038	F: AGGTTTAAGTTTCCCACG	685	55
MYO1038	R: CTTCCTCCTCTTATGTGC		
LRP414	F: TGGCTCACCAGTCAGGAA	764	55
LRP414	R: TACTCAGGCCGGAACAAG		
FREM225	F: CCATACTGAGCCATCCTA	688	55
FREM225	R: CCATCAGAACAACGAAAT		
FMN178	F: GCTCACGGACAGCTCTTG	382	55
FMN178	R: AGGCATAATGGAAGGCAC		
