# Supplementary material for: Identification of a HOXD13 variant in a Mongolian family with incomplete penetrance syndactyly by exon sequencing
Source: BMC Med Genomics. 2022 Oct 4;15:210. doi: 10.1186/s12920-022-01360-3 (PMC9533607; doi:10.1186/s12920-022-01360-3)
Supplement: Supplementary file 2 — Supplementary Material 2 [file 12920_2022_1360_MOESM2_ESM.pdf]

Additional file 2

A: 2%agarosegel electrophoresis was used to detect PCR products (M. DL 5000 DNA marker; subjects) B: Sanger sequencing validation results(Note: the gel figure was cropped; The original gel image is in Additional file3: Figure S2).

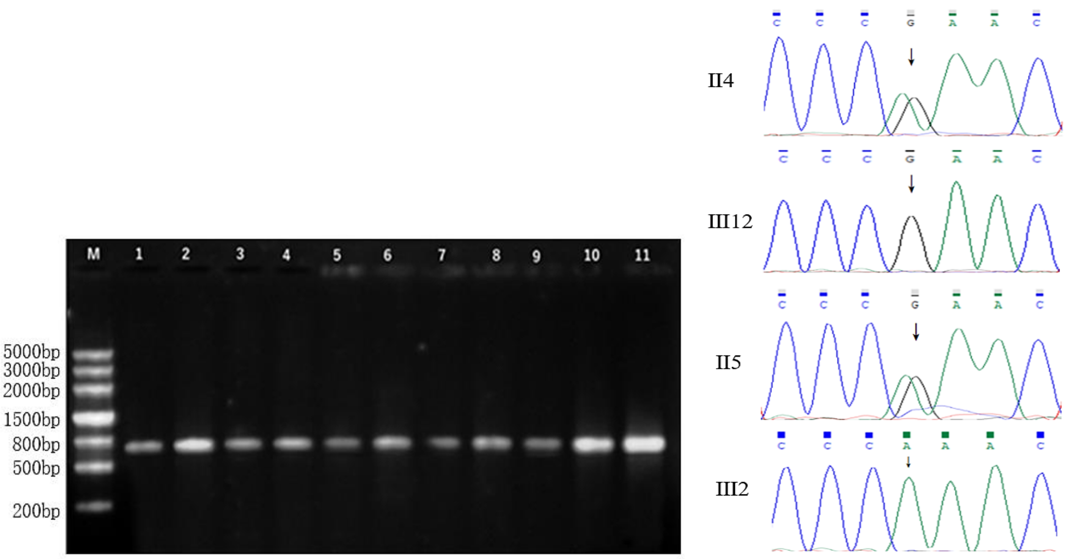

A B

A: *MYO10*: target fragment length 685bp.

B: II_4 proband is GA heterozygous; The carriers with incomplete penetrance of III_{12} has normal phenotype, wild type is GG homozygous; II_5 normal member, the genotype is GA heterozygous; III_2 normal member, the genotype is AA homozygous.

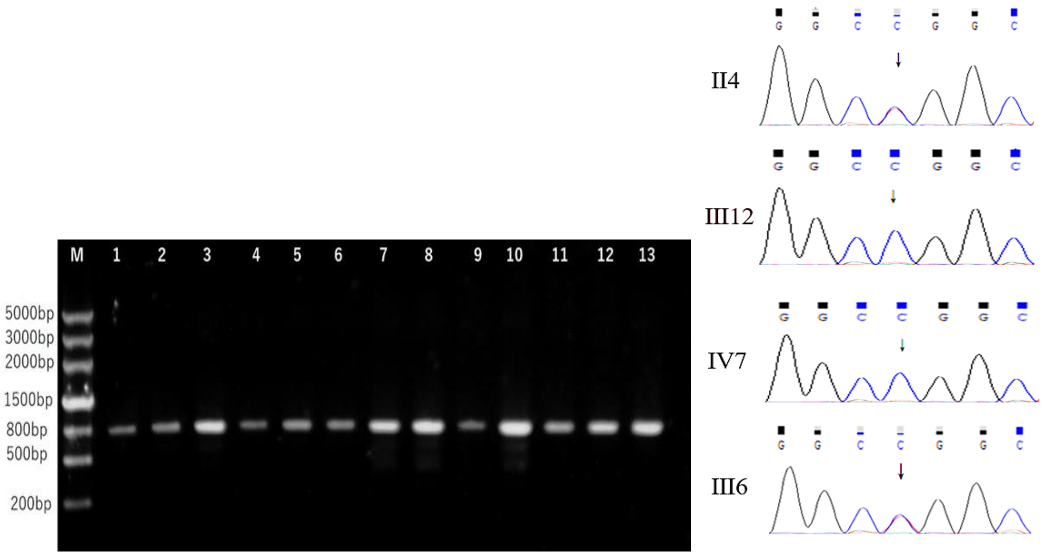

A B

A: *LRP4*: target fragment length 764bp.

B: II_4 proband is TC heterozygous; The carriers with incomplete penetrance of III_{12} has normal phenotype, wild type is CC homozygous; IV_7 patients, wild type is CC homozygous; III_6 normal

member, the genotype is TC heterozygous.

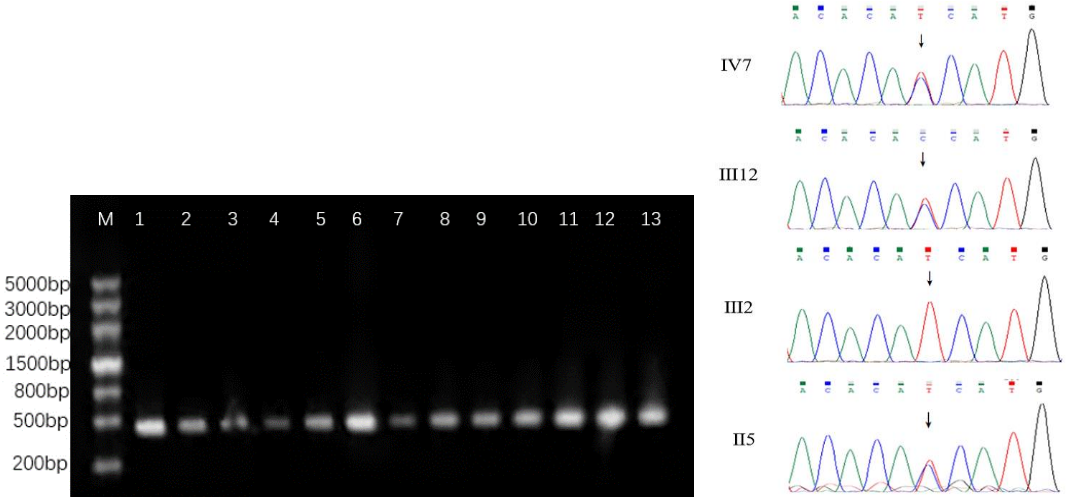

A B

A: *FBM2*: target fragment length 445bp.

B: IV_7 patients is TC heterozygous; The carriers with incomplete penetrance of III_{12} has normal phenotype, but the genotype is TC heterozygous; III_2 normal member, wild type is TT homozygous; II_5 normal member, the genotype is TC heterozygous.

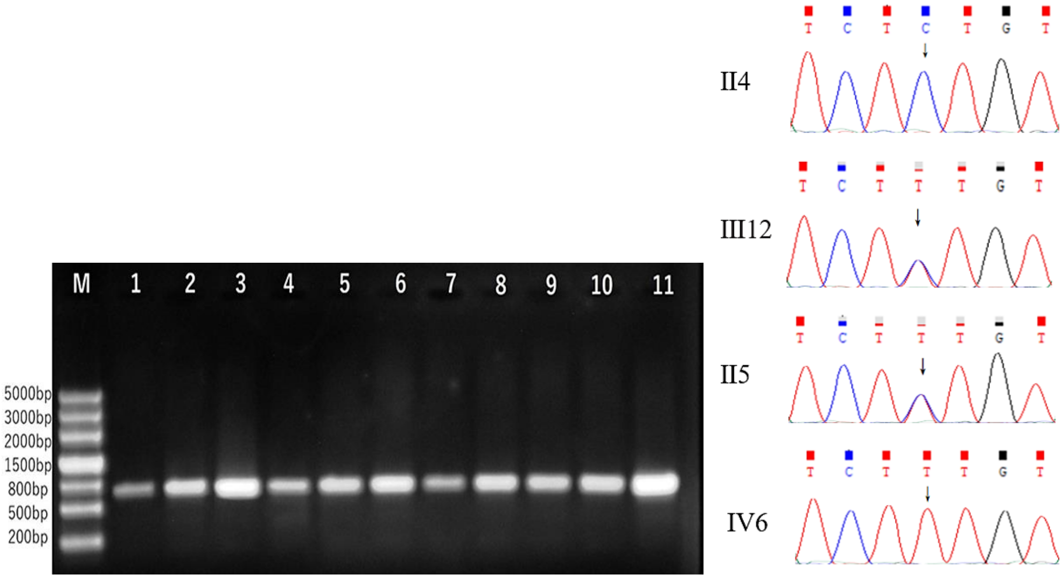

A B

A: *FREM2*: target fragment length 688bp.

B: II_4 proband is CC homozygous; The carriers with incomplete penetrance of III_{12} has normal phenotype, wild type is TC heterozygous; II_5 normal member, the genotype is TC heterozygous; IV_6 patients, wild type is TT homozygous.

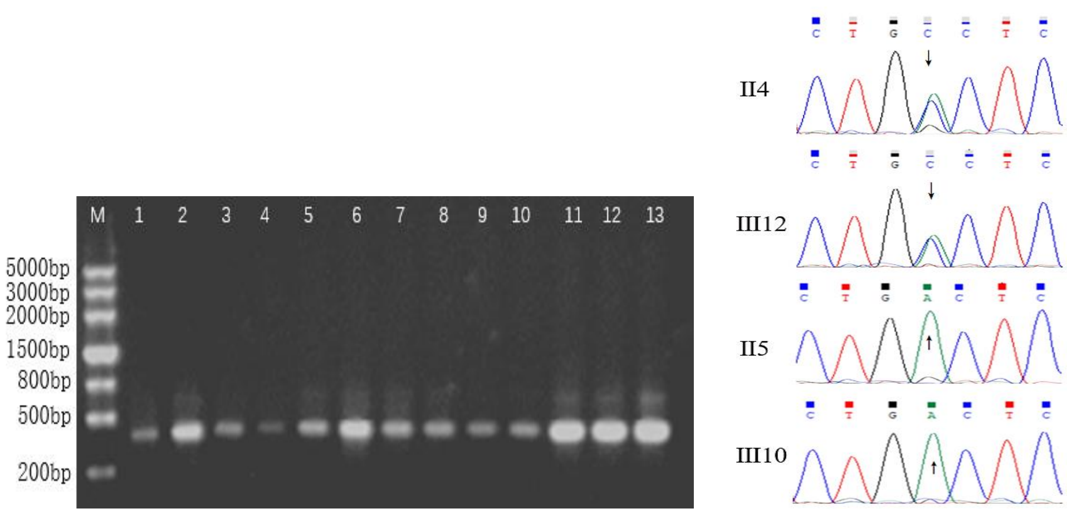

A B

A: *FMN1*: target fragment length 382bp.

B: II_4 proband is AC heterozygous; The carriers with incomplete penetrance of III_{12} has normal phenotype, but the genotype is AC heterozygous; II_5 normal member, genotype is AA homozygous; In patients with III_{10}, the wild type is AA homozygous.
