# Supplementary material for: Identification of a HOXD13 variant in a Mongolian family with incomplete penetrance syndactyly by exon sequencing
Source: BMC Med Genomics. 2022 Oct 4;15:210. doi: 10.1186/s12920-022-01360-3 (PMC9533607; doi:10.1186/s12920-022-01360-3)
Supplement: Supplementary file 3 — Supplementary Material 3 [file 12920_2022_1360_MOESM3_ESM.pdf]

Additional file3 FigureS2

In order to make the original image of gel clear and easy to distinguish, a high contrast method was adopted.

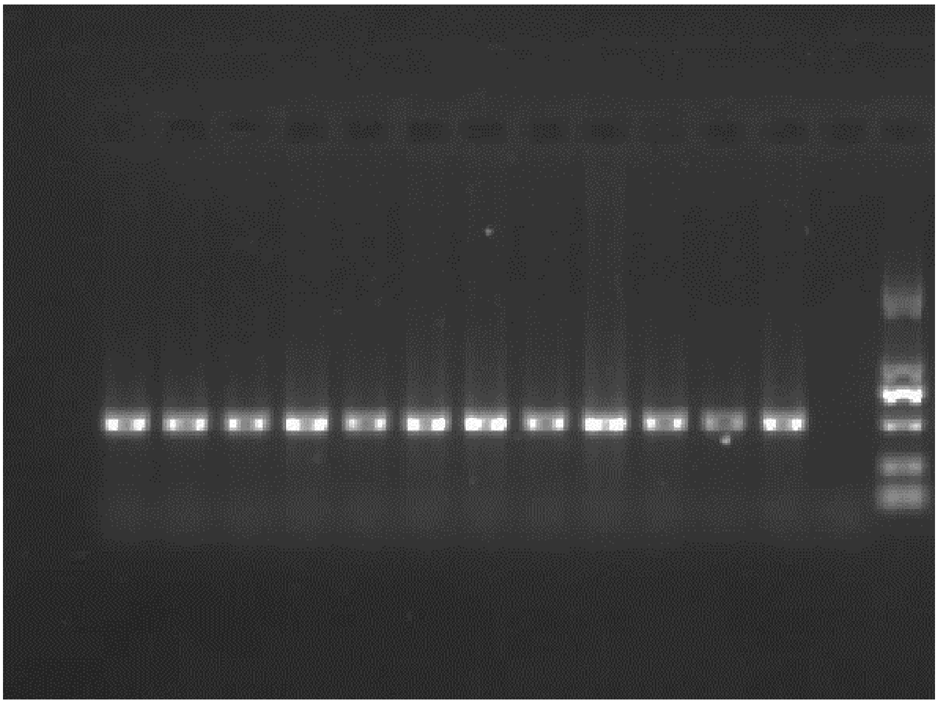

HOXD13 gene gel original image

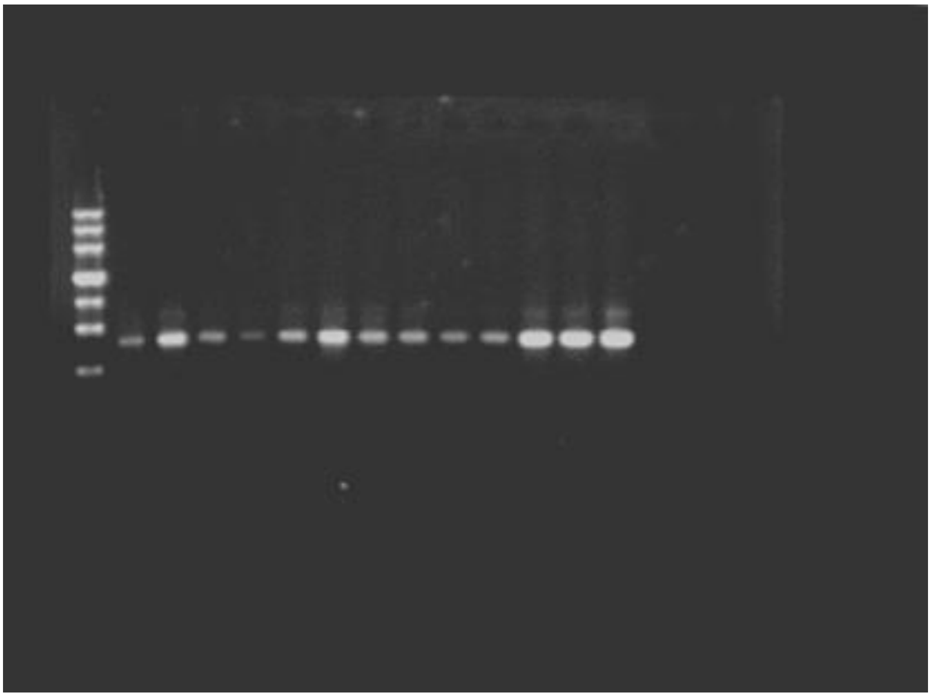

FMN1 gene gel original image

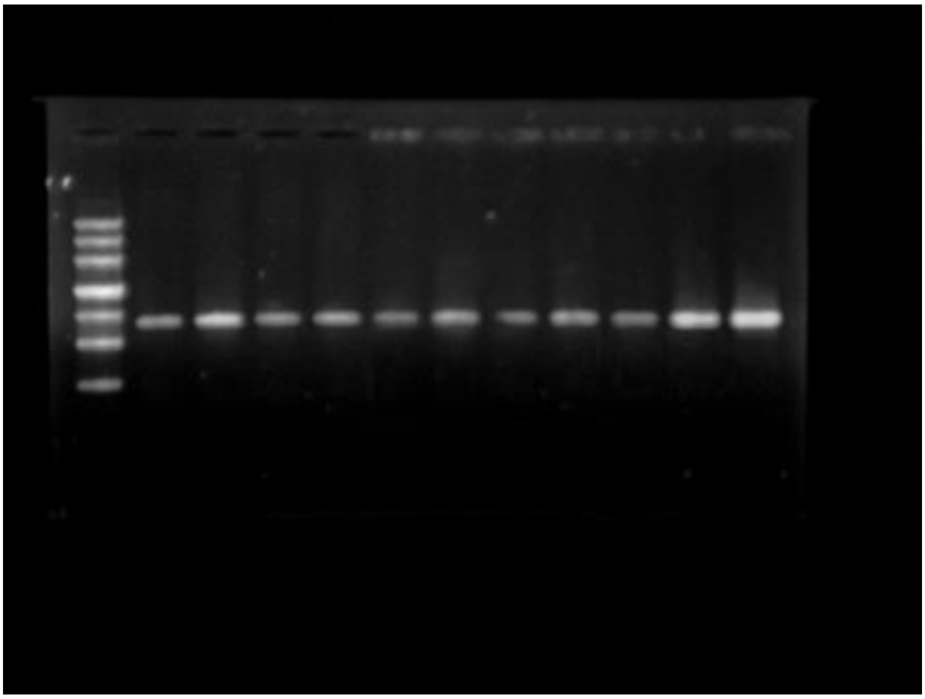

MYO10 gene gel original image

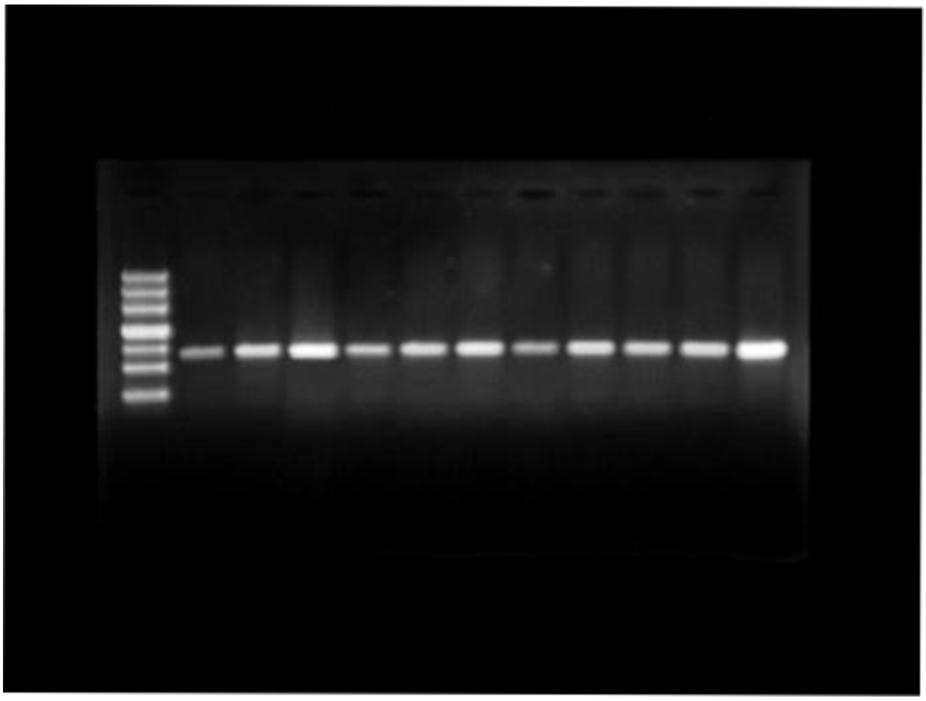

FREM2 gene gel original image

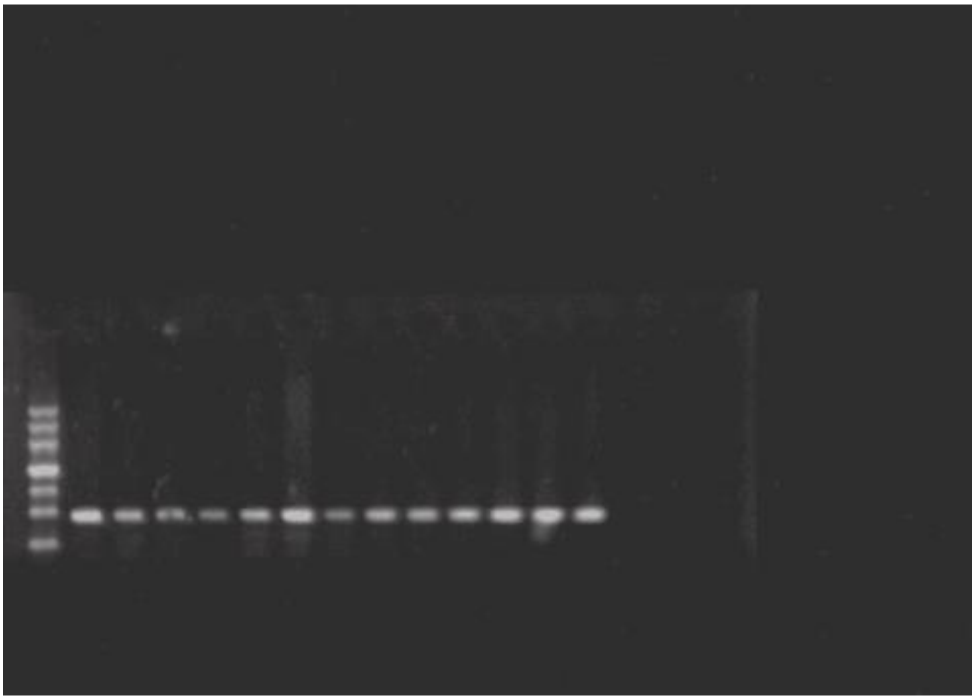

FBM2 gene gel original image

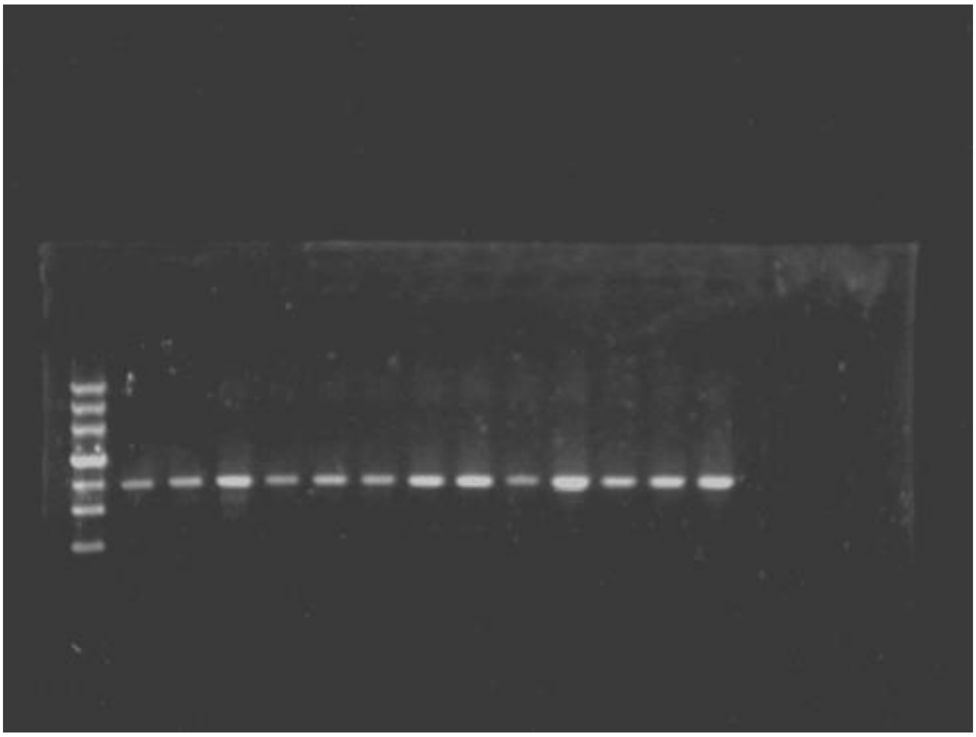

LRP4 gene gel original image
